# Rapid detection of cultivated truffle fungi (*Tuber*) from truffles and ectomycorrhizal root tips using loop-mediated isothermal amplification

**DOI:** 10.1128/aem.01559-26

**Published:** 2026-08-28

**Authors:** Alassane Sow, Andrew Cooper, Inga Meadows, Gregory Bonito, Matthew E. Smith

**Affiliations:** 1 Department of Plant Pathology, University of Florida 3463, Gainesville, Florida, USA; 2 Mountain Research Station, North Carolina State University 6798, Waynesville, North Carolina, USA; 3 Department of Microbiology, Genetics & Immunology, Michigan State University 3078, East Lansing, Michigan, USA; Royal Botanic Gardens Kew, Surrey, United Kingdom

**Keywords:** ectomycorrhizas, LAMP, truffle production, trufficulture, truffière, *Tuber aestivum*, *Tuber borchii*, *Tuber brumale*, *Tuber melanosporum*

## Abstract

Truffle fungi, such as *Tuber melanosporum* and *T. aestivum*, are prized luxury food products that have been historically cultivated in Mediterranean regions of Europe. Recent advances in truffle cultivation have led to the establishment of productive truffle orchards in North America, South America, China, and Australia. Despite these advances, a consistent issue has been the introduction and accidental cultivation of non-target truffle species. Non-target truffle species (e.g., *T. indicum* and *T. brumale*) often morphologically resemble target species (e.g., *T. melanosporum*) and may unintentionally be included as an inoculum and introduced to seedlings. Detecting inoculum and seedling contamination is difficult and often relies on inconsistent morphological analyses or labor-intensive and time-consuming PCR-based methods. Furthermore, quantitative PCR (qPCR) assays have only been developed for *T. melanosporum* and *T. aestivum,* leaving truffle growers without rapid tools to detect important contaminants such as *T. brumale*. Here, we develop a rapid loop-mediated isothermal amplification (LAMP) assay to detect three globally cultivated *Tuber* species (*T. melanosporum*, *T. borchii*, and *T. aestivum*) and the most common introduced contaminant species (*T. brumale*). The assays presented here are sensitive to at least 10^−3^ ng of DNA and are species-specific. These assays should be of special interest to both truffle orchard owners and truffle testing facilities given the low time commitment, high sensitivity, high specificity, and cost comparable to PCR and qPCR-based approaches.

**IMPORTANCE:**

Despite the global success of truffle cultivation, the accidental cultivation of undesirable truffle species has been a persistent issue. To address this issue, we developed four loop-mediated isothermal amplification (LAMP) assays for the rapid detection of *Tuber aestivum*, *T. borchii*, *T. brumale,* and *T. melanosporum* from ectomycorrhizal root tips and truffle fruiting bodies. These assays are alternatives to the widely used *T. aestivum* and *T. melanosporum* qPCR assays and are the first rapid detection assays for *T. borchii* and *T. brumale*.

## INTRODUCTION

Truffle fungi in the genus *Tuber* are prized gourmet foods that often sell for hundreds to thousands of dollars per pound (1, 2). Truffles have historically been cultivated in a select few Mediterranean regions in Europe, but increased global interest in truffles and advances in cultivation methods have led to the establishment of productive orchards in Australia, China, New Zealand, North America, and South America (2–7).

Despite the global success of *Tuber* cultivation, there are considerable obstacles for both new and established orchards to overcome. Notably, seedling and inoculum contamination is a pervasive issue and leads to the loss of thousands of dollars in investments (8, 9). Competing ectomycorrhizal (ECM) fungi can inhibit the successful mycorrhization of roots with target *Tuber* species. Common greenhouse contaminants include *Pulvinula convexella* and *Sphaerosporella brunnea* (10, 11). Inoculum and inoculated seedlings may also be contaminated by non-target *Tuber* pest species such as *Tuber brumale* (8, 9). *Tuber brumale* is endemic to Europe, and many orchards across the globe obtain their truffle inoculum from this region. Unfortunately, poor quality control and lenient testing requirements have led to the accidental introduction of *T. brumale* to Australia, Canada, New Zealand, and most recently, the United States (8, 12–14). Though some countries have since established testing requirements for seedlings and inoculum, contamination still persists, and requirements have yet to be established in the emerging North American truffle industry (8).

Detecting inoculum and seedling contamination is a difficult task, especially for those new to the practice of trufficulture. A combination of morphological and molecular-based methods are used to verify inoculation, morphotype ectomycorrhizas, and quantify colonization by the target *Tuber* species (15–17). Visual and morphological analyses of ectomycorrhizas rely on distinguishing the characteristic appearance of ECM root tip “morphotypes” or the notable cystidia (modified hyphae that emanate from the mantle) of some *Tuber* species (10). For instance, *T. melanosporum* cystidia form distinct right-angle branches and are typically longer than those of *T. brumale* (8, 18, 19). Differentiation based on morphological features alone, however, is often unreliable because of limited variation in spore traits or ECM root tip morphology. Additionally, some *Tuber* ectomycorrhizas may lack cystidia, and the morphotyping method requires extensive knowledge of mycorrhizal morphology and terminology. Molecular methods based upon PCR of the ITS rDNA barcode or quantitative PCR (qPCR) of species-specific sequences are the most reliable techniques (16, 17). However, these methods are generally inaccessible to the public because they require training and specialized laboratory equipment. There are pay-for-service testing labs that use molecular identification, but the services may be prohibitively expensive, and the results are often not available for several weeks. Additionally, qPCR assays (which require a specialized thermocycler to perform) have only been developed for *T. melanosporum* and *T. aestivum* (20, 21). Here, we address these issues by developing an accessible, cheap, and rapid molecular assay that can detect cultivated *Tuber* species from ECM root tips or fruiting bodies and can be performed by truffle growers, nurseries producing trees inoculated with truffles, and truffle testing labs.

Loop-mediated isothermal amplification (LAMP) is a rapid, colorimetric molecular method that has recently been used to detect fungi from plant tissues (22–24) and other substrates (25). Briefly, the LAMP method works by using four primers to detect six regions of a target sequence and synthesize complementary strands that form stem-loop structures. These loop structures are then used as templates for amplification (for a detailed description of the LAMP method, see Wong et al. [26]). The isothermal nature of LAMP removes the need for thermocyclers, while colorimetric reagents allow users to visualize reaction progress without using gel electrophoresis, spectrophotometers, or fluorometers. Eliminating the need for expensive lab equipment significantly reduces the cost required for detection of target DNA when compared to PCR or qPCR. Species-specific assays also allow users to accurately detect fungi in a sample without using sequencing technology, such as Sanger sequencing, that can further increase sample processing time and costs (23, 27, 28).

Although these attributes make LAMP generally more accessible than PCR, high-quality DNA is still required for LAMP assays. Common DNA extraction methods require the use of toxic chemicals, expensive pre-packaged kits, expensive specialized equipment, and can take hours or days to complete (29). Flinders Technology Associates (FTA) card and Tris-NaOH based methods, however, are both nontoxic, relatively inexpensive techniques that require minimal technical expertise and do not require the use of specialized equipment (30). The use of FTA cards is straightforward such that people without a technical background can readily use them to stabilize DNA from samples rather than sending fresh root tips or truffle specimens to diagnostic labs, thus removing the need for expensive expedited and refrigerated shipping. Tris-NaOH DNA extractions also require minimal technical knowledge and equipment, are inexpensive, and can reliably extract DNA from both fresh and dried tissue (31).

Here, we develop species-specific and sensitive LAMP assays for the rapid detection of four economically significant European *Tuber* species; *Tuber aestivum*, *T. borchii*, *T. brumale,* and *T. melanosporum*. We demonstrate that the assays can be reliably completed in less than an hour. We also demonstrate the efficacy of both FTA cards and Tris-NaOH-based methods for the reliable extraction of DNA from truffles and ECM root tips for LAMP reactions. We anticipate that our rapid and accessible approach will reduce testing time, increase the adoption of testing among truffle growers, and reduce contamination in truffle orchards. We hope that the development of these new tools may also encourage the establishment of testing services in the United States and other emerging markets.

## MATERIALS AND METHODS

### Identification of unique marker regions and LAMP primer design

We downloaded the assembled genomes of our target species (*Tuber aestivum*, *T. borchii*, *T. brumale, and T. melanosporum*) and outgroup species (*Tuber magnatum, Tuber indicum,* and *Sphaerosporella brunnea*) from the NCBI database (Table S1). These genomes were generated in previous studies and were sequenced from cultures (*T. melanosporum*, *T. borchii, T. indicum,* and *S. brunnea*) and fruiting bodies (*T. brumale*, *T. aestivum,* and *T. magnatum*) using both long- and short-read sequencing technologies (32–35). We chose these outgroup species because they are close relatives to our target species; *T. magnatum* is a close relative of *T. aestivum,* while *T. indicum* is a close relative of *T. brumale* and *T. melanosporum* and is morphologically similar to both species (36). We chose *S. brunnea* as an outgroup because it is a common ectomycorrhizal contaminant that we expect to co-occur with *Tuber* (10, 37).

Using the program fur (38), we identified unique regions from each target genome. We used seqkit v2.4.0 and bioawk v1.0 to remove short (<300 bp) and long (>2,000 bp) sequences and sequences with degenerate bases, soft masked regions, and low GC content (≤40%) (39, 40). Examples of data and code used in this pipeline are available at https://github.com/trufflesmith/TruffleSmithLab/. To ensure that the identified regions were unique, we performed BLASTx and BLASTn searches of the regions using the whole-genome shotgun contig database restricted to fungal sequences, the core nucleotide database, and the RefSeq Genome database. We selected marker regions that had no BLAST hits for LAMP primer design.

We designed LAMP primer sets for each unique region using the NEB primer design tool v 1.5.1 (https://lamp.neb.com/#!/). A complete LAMP primer set consists of two inner primers and two outer primers. However, two additional loop primers can be designed that reduce the reaction time and increase the specificity of an assay (41). To guarantee rapid amplification, we preferentially selected primer sets that included both loop primers for a total of six primers per species-specific assay. Primers were synthesized by Integrated DNA Technologies (Newark, NJ, USA).

### DNA extraction for LAMP reactions

To test the accuracy, precision, and sensitivity of each LAMP primer set, we extracted DNA from fresh and dried truffle specimens using a modified CTAB extraction method, as described by Lemmond et al. (42) and Fauchery et al. (43). To determine the concentrations of DNA, we used the Qubit 4 Fluorometer and Qubit dsDNA BR assay kit (Thermo Fisher Scientific, Waltham, MA, USA).

To demonstrate the usefulness of our methods for testing truffles and ECM roots, we also extracted DNA using the Tris-NaOH “rapid extraction” method and FTA cards. To extract DNA using the Tris-NaOH rapid extraction method, we placed small (1–5 mg) pieces of dried truffles into 30 μL of Tris-NaOH buffer (1 mL/L of 1 M Tris, 0.186 g/L KCl, and 37 mg/L EDTA, brought to pH 9.5–10.0 with 1 M NaOH) and homogenized the material with a sterilized metal needle. Following homogenization, we incubated the mixture at 95°C for 10 min and then added 60 μL of 3% bovine serum albumin (BSA). We also stabilized DNA from ECM root tips and truffles using FTA cards and eluted DNA using the Zymo Research Quick-DNA Fungal/Bacterial MiniPrep Kit (Zymo Research Corporation, Irvine, CA, USA). Briefly, we placed about 20 ECM root tips in the pre-labeled FTA card circles before folding the lid of the card over the samples and thoroughly smashing the root tips. We then removed the ECM root tips and allowed the cards to dry for 1 h before storing at room temperature or proceeding directly with DNA extraction. We extracted DNA from one to three hole-punched sections (6 mm diameter each) of FTA cards following the instructions provided in the Zymo kit. Lastly, to determine the usefulness of our methods for testing for the presence of *Tuber* species in soils, we extracted DNA from soil collected from productive truffle orchards using either the Omega Bio-tek E.Z.N.A. Soil DNA kit (Omega Bio-tek Inc, Norcross, GA, USA) or the Qiagen PowerSoil Pro Kit (Qiagen, Venlo, Netherlands).

We confirmed the identity of *Tuber* from the extracted material (truffles or ECM root tips) by amplifying and sequencing the ITS region using combinations of forward primers ITS1F or ITS1 and reverse primers LR3 or ITS4 (44, 45). PCR amplification was confirmed using gel electrophoresis followed by Sanger sequencing at Eurofins Genomics (Louisville, KY, USA). We trimmed low-quality regions of sequences using Geneious Prime v2023.1.2. We confirmed the presence of *T. aestivum* and *T. melanosporum* in soils and root tips using species-specific qPCR assays (20, 21). We also confirmed the presence of *T. aestivum, T. borchii,* and *T. melanosporum* in soils based on recently published ITS metabarcoding data from soils in Australia and Michigan, USA (37, 46).

Altogether, the samples used to test the efficacy of our LAMP assays consisted of truffles collected in North American, Chinese, and European truffle orchards, truffles collected in Japanese forests, ECM root tips from North American orchards, and soils from North American and Australian truffle orchards (Table S2). Many of the samples from North American truffle orchards were provided to us by truffle growers who used the truffle testing services of the North Carolina State Truffle Lab (Waynesville, NC, USA). We have anonymized the collection information for these samples (Table S2). All of the dried truffle specimens used in this study are accessioned in the Florida Museum of Natural History Fungal Herbarium, the Michigan State University Herbarium, and the Kanagawa Prefectural Museum of Natural History Herbarium (Japan) (Table S2).

### LAMP assay specificity, accuracy, species specificity, and limits of detection

For each LAMP assay, we mixed 1–5 μL of DNA with 12.5 μL of WarmStart Colorimetric LAMP 2× Master Mix (New England Biolabs, Ipswich, MA, USA), 4–9 μL of molecular-grade water, and 2.5 μL of LAMP primer mix (16 μM forward inner primer, 16 μM backward inner primer, 2 μM F3, 2 μM B3, 4 μM Loop F, and 4 μM Loop B). For negative controls, we replaced DNA with 1 μL of sterile molecular-grade water. Reaction mixes were incubated at 65°C for 30 min and up to 1 h if the color change was not robust. Reaction progress was verified by visual assessment (i.e., positive reactions turned yellow while negative reactions remained pink) and subsequent gel electrophoresis.

To test the species-specificity of each primer set, we ran triplicate reactions with 10 ng of DNA from each of our target species (*T. aestivum, T. borchii, T. brumale,* and *T. melanosporum*), truffle species common in North American orchards (*T. canaliculatum*, *T. cumberlandense,* and *T. lyonii*), truffle species we do not expect to be present in North American orchards (*T. himalayense*, *T. indicum*, *T. longispinosum*, and *T. oregonense*), and an ectomycorrhizal fungus that is a common contaminant in truffle orchards (*Sphaerosporella brunnea*).

To identify the limit of detection of each primer set, we ran triplicate reactions with 1:10 serial dilutions of DNA from 10 ng/μL to 10^−6^ ng/μL. It is important to demonstrate the ability of our assay to detect DNA from target *Tuber* species when multiple *Tuber* species are present (for instance, when DNA is unintentionally extracted from multiple ECM root tips colonized with different *Tuber* species). We designed an experiment wherein we mixed 1 μL of 10 ng/μL DNA from three of the four species of interest (*T. aestivum, T. borchii, T. brumale,* and *T. melanosporum*) with 1 μL of DNA from the target species that we serially diluted from 10 ng/μL to 10^−3^ ng/μL (e.g., increasingly reduced amounts of target DNA versus total nontarget DNA from other *Tuber* species). We used 10 ng of target DNA as a positive control and water as a negative control. This experiment was repeated three times.

Finally, to demonstrate that our target sequences are conserved within each target species, we tested the ability of each primer set to detect specimens of the target *Tuber* species collected from different truffle orchards across the United States, Europe, and Australia (Table S2). In total, each assay was tested on at least 10 samples of its respective target *Tuber* species and three sample types (root tips, soil, and truffles) (Table S2). Here, we present a primer set for each species that was consistently species-specific, sensitive to low amounts of target DNA, and that reliably amplified DNA in 30 min.

## RESULTS

**Fig 1:**
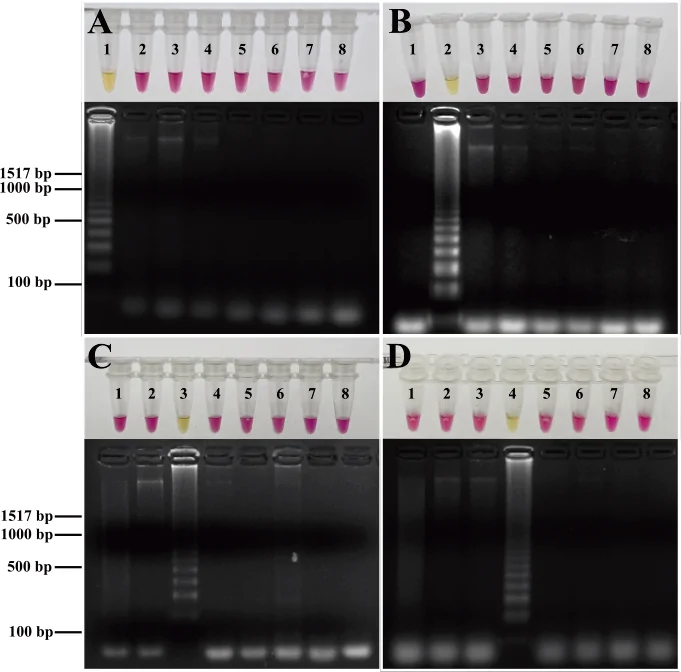
Examples of LAMP detection of *Tuber melanosporum* (**A**), *T. brumale* (**B**), *T. borchii* (**C**), and *T. aestivum* (**D**). Yellow reactions are positive, and pink reactions are negative. In each image: 1 = *Tuber melanosporum* (FLAS-F-72933), 2 = *T. brumale* (FLAS-F-71077), 3 = *T. borchii* (FLAS-F-71198), 4 = *T. aestivum* (FLAS-F-71186), 5 = *T. canaliculatum* (FLAS-F-72952), 6 = *T. lyonii* (BL94), 7 = *T. indicum* (KPM-NC0031719), and 8 = water. LAMP reactions were run on 2% agarose gels at 100 V for 30 min. Note that large bands in wells 2, 3, and 4 are fragments of genomic DNA.

We identified 40 unique *T. aestivum* sequences, 61 unique *T. borchii* sequences, 37 unique *T. brumale* sequences, and 35 unique *Tuber melanosporum* sequences for LAMP primer design. Of these, we were able to design complete LAMP primer sets with loop primers for 14 *T. aestivum* sequences, 17 *T. borchii* sequences, 14 *T. brumale* sequences, and five *T. melanosporum* sequences. We tested the efficacy of these primer sets and present here an assay for each species that was species-specific, sensitive to 10^−3^ ng of DNA, amplified target DNA within 30 min, and detected the target species from multiple orchards and specimens (Fig. 1; Table 1; see Table S3 for the target sequences used for primer design).

**TABLE 1:** Limits of detection, sensitivity, specificity, and accuracy of LAMP primer sets used for the rapid detection of *Tuber aestivum, T. borchii, T. brumale,* and *T. melanosporum* from truffles and ectomycorrhizal root tips^*e*^

Target species	Primer	Primer sequence (5′ → 3′)	Limit of detection (ng of DNA)	Specificity and 95% CI^*a*^^,^^*d*^	Sensitivity and 95% CI^*b*^^,^^*d*^	Accuracy and 95% CI^*c*^^,^^*d*^
*Tuber aestivum*	F3	CGAGTAGTTGCGTTGGAGAT	10^−3^	0.96 (0.82–0.99)	1.00 (0.78–1.00)	0.95 (0.85–0.99)
	B3	CCGAAAGGGGTGCCAATT				
	FIP	TTCCGCGCCTCCCACTAAGAATGCGAGGTAATCGCCTTTACG				
	BIP	GCACCTCGGTATCGCTGCTGGGCGTGGAGAGAGGAGTA				
	LF	CCCCCGTCCCCTGTGTTAAA				
	LB	CGTATCATAGAGGCGCCTTTGAG				
*Tuber borchii*	F3	CATCACAGACGAGCAGCAG	10^−3^	1.00 (0.88–1.00)	1.00 (0.78–1.00)	1.00 (0.92–1.00)
	B3	TCCTACCTCACTCCACCG				
	FIP	CGGCAGCCTCAAAAACAAACGATTACAGCTCCTGCAGGGTAT				
	BIP	TACATTCGGGCGATTTGGGTCCCCATCAAGACGGCATCTCC				
	LF	TCTTACCACTGCCATACCGTAGA				
	LB	AGGCGCTGGAAGATCTTGGT				
*Tuber brumale*	F3	CGGTATCTGCTGCTCACAAT	10^−3^	0.96 (0.82–0.99)	1.00 (0.78–1.00)	0.95 (0.85–0.99)
	B3	CGGCACTCGGAGTGAGAA				
	FIP	TGCTGGCTTAGGTGCCTAGGTAACACACACAGACACACACTT				
	BIP	TGTCGAACCCCACTGCTTGTTGAATCGGCTGGTTGTTGGTT				
	LF	GACTTCGGATACCGTAGCAAGG				
	LB	TTCGTCACCCCATCCGATCA				
*Tuber melanosporum*	F3	CTCAATGGTGGCACGAGAT	10^−3^	1.00 (0.88–1.00)	1.00 (0.78–1.00)	1.00 (0.92–1.00)
	B3	CTCGGATACCCGTCAACCTA				
	FIP	CGCCTTCGACATCTTCCCTTGAGGATCATGGCATTGGCGT				
	BIP	ATGATACAGCGTCCCCCCACACCAACTCAGGAAGTGAACGT				
	LF	TCTGCATTTAGCTTCTGTGCTCC				
	LB	ACGGAGGATAAATCTCAAACTTGGA				

^*a*^ Specificity was calculated by dividing the number of negative reactions by the total number of negative controls (*n* = 30; 15 negative controls were water and 15 were non-target DNA).

^*b*^ Sensitivity was calculated by dividing the number positive reactions by the total number of samples with target DNA (*n* = 15).

^*c*^ Accuracy was calculated by dividing the number of true positive and true negative reactions by the total number of reactions (*n* = 45).

^*d*^ Note that we did not include results from our soil extraction experiments in this calculation.

^*e*^ For the target sequences used for primer design, see Table S3. F3, forward outer; B3, reverse outer; FIP, forward inner primer; BIP, backward inner primer; LF, forward loop primer; LB, backward loop primer.

Each assay was sensitive to the concentrations of DNA expected from all of the different extraction methods (Table 1; Table S2). The CTAB extraction method yielded 0.3 ng/μL–48 ng/μL of DNA from dried truffles, the FTA card extraction method yielded 0.1 ng/μL–3 ng/μL of DNA from ECM root tips and fresh truffles, and the Tris-NaOH extraction method yielded 3 ng/μL–7 ng/μL of DNA from dried truffles. In reactions mixed with 30 ng of nontarget *Tuber* DNA, each assay was sensitive to as little as 10^−2^ ng of target DNA. We note that in one replicate, neither the *T. aestivum* nor *T. brumale* assays detected the DNA at the lowest concentration (10^−2^ ng of target DNA) in the mixed samples, even after incubating the reactions for 1 h.

Each LAMP assay presented here reliably detected DNA extracted from both ECM root tips and truffles. However, when testing the ability of the assays to detect target DNA in soil, we did not detect *T. borchii* and *T. aestivum* from some samples irrespective of the amount of DNA added to those reactions (1–5 μL) or incubation time (up to 1 h) (Table 2). The *T. melanosporum* assay performed better and consistently detected target DNA from all 10 soil DNA samples (Table 2).

**TABLE 2:** Comparison of LAMP and qPCR detection of *Tuber melanosporum* and *T. aestivum* from soils collected from orchards within the United States

Species	Soil sample^*a*^	Detection method	
		LAMP^*b*^	qPCR^***b***^
*Tuber aestivum*	23-287	+	+
*Tuber aestivum*	23-288	+	+
*Tuber aestivum*	23-302	+	+
*Tuber aestivum*	23-223	−	+
*Tuber aestivum*	25-214	+	+
*Tuber melanosporum*	22-367	+	+
*Tuber melanosporum*	22-368	+	+
*Tuber melanosporum*	22-369	+	+
*Tuber melanosporum*	23-220	+	+
*Tuber melanosporum*	24-005	+	+

^*a*^ All samples were provided by truffle growers using the North Carolina State University Truffle Lab services. For more information about each sample and other samples tested, see Table S2.

^*b*^ The LAMP primers described in this paper were used for LAMP detection, and the species-specific qPCR primers designed by Parladé et al. (21) and Gryndler et al. (20) were used for qPCR detection.

## DISCUSSION

Here, we present the first rapid, species-specific assays designed for the detection of *T. borchii* and the common contaminant, *T. brumale*. To our knowledge, only members of the *Tuber brumale* clade A, the most common subgroup in western Europe, are present in North American orchards (8, 47). We note that the *Tuber brumale* genome used to develop the LAMP assay was collected from France and is a member of this clade (Fig. S1). Additionally, all of the *T. brumale* collections used to test the specificity of the primers (e.g., FLAS-F-71077) are also members of clade A (Fig. S1) (8). Because no specimens of *Tuber brumale* clade B were available to us for testing and there is not a publicly available genome for *Tuber brumale* clade B, we only tested the LAMP assay on members of clade A. Therefore, we cannot be certain that our *T. brumale* primers can be used to detect members of the putatively distinct phylogenetic species in *Tuber brumale* clade B (47).

We are confident, however, that our *T. aestivum* primers can be used to detect both *T. aestivum* and its long-debated synonymous species, *T. uncinatum* (48). Some nurseries sell seedlings inoculated with *T. uncinatum* because the truffles identified as *T. uncinatum* are regarded by some people as more flavorful, and thereby more valuable, than *T. aestivum* (49). However, several studies have used multigene phylogenies and coalescent analyses to demonstrate that *T. aestivum* and *T. uncinatum* are conspecific (49–52). Accordingly, we did not distinguish between *T. aestivum* and *T. uncinatum* samples when testing the efficacy of our *T. aestivum* assay. Recently, Riccioni et al. analyzed the genetic structure of *T. aestivum* across its native range and documented high haplotype diversity and the presence of a potentially cryptic species in Turkey (53). Because we did not test our assay on *T. aestivum* collections from Turkey, we cannot be certain that our assay would detect Turkish haplotypes. However, most nurseries that source seedlings for the global cultivation of *T. aestivum* are based in western Europe, and we demonstrate that our assay can detect *T. aestivum* from several independent truffle orchards in California, Oregon, and North Carolina (Table S2).

Although we inconsistently detected *T. aestivum* and *T. borchii* from soils, we detected *T. melanosporum* in all soil samples. This was likely due to the characteristically high abundance of *T. melanosporum* mycelium in the brûlé area (46). This habit has not been documented in *T. aestivum* nor *T. borchii,* and the inconsistent detection of these species may be due to low mycelial abundance in some samples or the presence of soil inhibitors that impact the LAMP assays. The exact cause of inconsistent detection from soil is unclear as neither increasing the amount of DNA (to help with low template concentration) nor diluting the DNA (to reduce the effects of soil inhibitors) enabled detection. When testing *T. melanosporum* and *T. aestivum* truffles and mycorrhized root tips, our assays performed similarly to qPCR (Table 2). However, based on the inconsistent soil detection results, we do not recommend using our assays to detect *Tuber* DNA from soil and instead recommend the widely used qPCR assays for these species as more consistent alternatives (Table 2) (20, 21).

Although the assays presented here were species-specific, during our testing, we observed two false-positive reactions when using the *T. brumale* and *T. aestivum* assays. We attribute the false positives to carryover contamination from pipettes used for both reaction mixing and pipetting amplified DNA. We were able to distinguish carryover contamination from cross-reactivity because after discarding the contaminated DNA and using a new extraction from the same truffles, no false positives were observed. Because the LAMP “loop” structures are stable and present in large amounts, they can contaminate reactions and lead to false-positive results if caution is not used during reaction mixing (54). To reduce carryover contamination, we recommend using filtered pipette tips, cleaning pipettes and work surfaces with bleach after each use, and using LAMP master mixes supplemented with dUTP and uracil-DNA glycosylase so as to enzymatically digest amplicons (54).

In conclusion, we present four species-specific LAMP assays for the rapid detection of *Tuber* from ECM roots and truffles. Our assays may be of particular interest to truffle testing labs and truffle orchard owners because the methods cost approximately the same as traditional PCR-based protocols but produce results considerably faster (Table 3). Additionally, we developed assays for the rapid detection of two truffle species for which there are no qPCR-based protocols, *T. borchii* and *T. brumale*. When using our LAMP assays in conjunction with the Tris-NaOH fast extraction method, many samples can be extracted and tested within an hour. Example uses for our assays in trufficulture are as follows: (i) confirming the species identity of truffles or truffle products used for inoculum, (ii) determining whether seedlings are mycorrhized with the correct *Tuber* species prior to planting in the orchard, (iii) determining whether *Tuber* has persisted on ECM roots 1–2 years after planting, and (iv) detection of the contaminating species, *Tuber brumale*, in an orchard from truffles or ECM root tips (8, 55). We hope the rapid and sensitive assays presented here will lead to improved quality control in emerging truffle markets and reduce the introduction of *Tuber brumale* and other nontarget species to truffle orchards globally.

**TABLE 3:** Comparison of the estimated time and reagent costs of LAMP, PCR, and qPCR detection of *Tuber^a^*

	Cost or time per reaction		
	LAMP	PCR	qPCR^*b*^
Reagents/services			
Master mix	$2.00^*c*^	$1.70^*d*^	$0.65^*e*^
Primers^*f*^	$0.06	$0.02	$0.02
Sequencing	–^*g*^	$0.33^*h*^	–
Estimated cost per reaction	$2.06	$2.02	$0.67
Protocol			
Reaction mixing	15 min	15 min	15 min
Incubation	30 min	≥1 h 30 min	≥1 h 30 min
Amplification confirmation^*i*^	1 min	30 min	1 min
Sequencing	–	1–2 days	–
Total time	45 min	1–2 days	≥1 h 45 min

^*a*^ For consistent comparisons of reagent costs, the cost per reaction of each reagent/service was calculated from the price of 500 reactions, and the same supplier was used for each reagent. Because quotes for thermocyclers vary widely, we do not include the cost of equipment here; however, it is important to note that PCR and qPCR both require thermocyclers that can cost several thousand dollars, whereas LAMP can be performed with heating blocks or dry baths that only cost a few hundred dollars.

^*b*^ The price of running standard curves is not included in our estimate.

^*c*^ M1800L NEB WarmStart Colorimetric LAMP 2× Master Mix (DNA & RNA).

^*d*^ M0541L NEBNext High-Fidelity 2× PCR Master Mix.

^*e*^ M3003L NEB Luna Universal qPCR Master Mix.

^*f*^ Eurofins Genomics Custom DNA Oligo in Tubes.

^*g*^ – indicates that the service or product is not used for the corresponding method.

^*h*^ Eurofins Genomics Prepaid Plate DNA Kit.

^*i*^ Meaning visual verification for LAMP, gel electrophoresis for PCR, and verification using analysis software for qPCR.

## Data Availability

The code used to generate these data and the associated sequences can be found on GitHub at https://github.com/trufflesmith/TruffleSmithLab/.

## SUPPLEMENTAL MATERIAL

Fig. S1; Tables S1 and S3 aem.01559-26-s0001.docx Phylogenetic tree and sequences.

Table S2 aem.01559-26-s0002.xlsx Sample metadata and testing results.

ASM does not own the copyrights to Supplemental Material that may be linked to, or accessed through, an article. The authors have granted ASM a non-exclusive, world-wide license to publish the Supplemental Material files. Please contact the corresponding author directly for reuse.
