# Supplementary material for: Rapid detection of cultivated truffle fungi (Tuber) from truffles and ectomycorrhizal root tips using loop-mediated isothermal amplification
Source: Appl Environ Microbiol. 2026 Aug 28;92(9):e01559-26. doi: 10.1128/aem.01559-26 (PMC13599548; doi:10.1128/aem.01559-26)
Supplement: Supplementary file 1 [file aem.01559-26-s0001.docx]

**SUPPLEMENTARY FIGURES –**

**Figure S1.** Maximum likelihood phylogeny of ITS DNA sequences showing placement of the *Tuber brumale* reference genome (purple) and a *T. brumale* sample used to test LAMP assay specificity (blue) in *T. brumale* clade A.

**SUPPLEMENTARY TABLES –**

**Table S1.** Whole genome sequences used to identify unique sequence regions for LAMP primer design.

| **Species** | **Isolate** | **GenBank** | **Citation** |
| --- | --- | --- | --- |
| *Sphareosporella brunnea* | Sb_GMNB300 | GCA_008704415 | (Benucci et al. 2019) |
| *Tuber aestivum* | Montdore | GCA_900010015 | (Murat, Payen, et al. 2018) |
| *Tuber borchii* | Tbo3840 | GCA_003070745 | (Murat, Kuo, et al. 2018) |
| *Tuber brumale* | 140227-1 | GCA_014065205 | (Morin et al. 2021) |
| *Tuber indicum* | Tubin_1 | GCA_014065215 | (Morin et al. 2021) |
| *Tuber indicum* | Tin1 | GCA_006112555 | - |
| *Tuber magnatum* | 091103-1 | GCA_003182015 | (Murat, Payen, et al. 2018) |
| *Tuber melanosporum* | Mel28 | GCA_000151645 | (Martin et al. 2010) |

**Table S2.** Samples used to test specificity and sensitivity of LAMP primer sets. A ‘+’ indicates that the corresponding sample tested positive with one of the LAMP assays. The truffles used to test the LAMP assays are accessioned in the Florida Museum of Natural History (FLAS-F) Fungal Herbarium and the Kanagawa Prefectural Museum of Natural History Herbarium (KPM).

(See attached excel sheet)

**Table S3.** Target sequences used for LAMP primer design

| **Species** | **Target Sequence** |
| --- | --- |
| *Tuber aestivum* | >Tuber_aestivum_Montdore_LAMP  TAGTACGAGTAGTTGCGTTGGAGATGCGAGGTAATCGCCTTTACGCCTTTAACACAGGGGACGGGGGGATTCTTAGTGGGAGGCGCGGAAAGCACCTCGGTATCGCTGCTGCATACCAGTATCGTATCATAGAGGCGCCTTTGAGCGTTTGATTACTCCTCTCTCCACGCCAAGAATTGGCACCCCTTTCGGGATCGCACATACCGTTTGTTGCTAGCCTATAACGGGAGAACGGATGATAATTCTTGGCTTGGAGGGGTGGGCGCGGACTAGTGCGTCTTGGCGGTACAGAATACCGTAGAAGTTTAGCAGGAGATAGTCTAACCTCAATTTTCCCTGTCATCCTTCGCGAACCGAGGCAACTCAGGGTTTCTCTTATTCCCGTGTGGGGCATTTTCATCTCCCAGCTATTTTGTTTTTTCCCTCACACCCGACCCTGGAGCTAGGAACCGGCCGTGTTTCCTGGCTCGAATTCCTTCCACCTTCTTTCTCTGTTCCTCGGGGAAGCCGACAGGCACCG |
| *Tuber borchii* | >Tuber_borchii_Tbo3840_LAMP  ATTGTGTGAATTTAAGAGAGCGGTACCCTACGAATCGTTGGTGCGAGTGCTAGCAGGACCAGCATCACAGACGAGCAGCAGTTACAGCTCCTGCAGGGTATTCTACGGTATGGCAGTGGTAAGAATCAAGACATGTTCGTTTGTTTTTGAGGCTGCCGCAGCTACCTCTGGAAATACATTCGGGCGATTTGGGTCCGAGGCGCTGGAAGATCTTGGTACGGGAGATGCCGTCTTGATGGCGGTGGAGTGAGGTAGGATCTCTTGAGATAATAGTACTGAATGAACTCCAGCTTACCAAATGCAGAGTACCGGACAGTGTTCTTATGAGATTGGAAATTGCTAGAGTGTTACTGGTACTCGAATAATTTGGTATTTTGGGGGAAGTGATTGTACGAGCACTTGGATGAATACAGATACTGGTAACTTTATATAGATTGCAGGCTGATTAGGATCGGTTTGACCCTCTTGGCTCATTGTTATCCTAGCGGCCCGGTCACTCCGG |
| *Tuber brumale* | >Tuber_brumale_140227-1_LAMP  CATTTACCTTTACTACACCTAGTGCCCATAATTTACAGTACCTTAGAGTACGGTATCTGCTGCTCACAATATTGTCCTCGCAAAATGACACACACAGACACACACTTACCTTGCTACGGTATCCGAAGTCAGAATTCTGCCTCCTTTTACCTAGGCACCTAAGCCAGCACAACCTGACTTGTCGTATACTACGGTATTATAATTACTTGTCGAACCCCACTGCTTGTTTTCGTCACCCCATCCGATCAAACCAACAACCAGCCGATTCGAGAGCCCGGACAATATTCTCACTCCGAGTGCCGGTATTT |
| *Tuber melanosporum* | >Tuber_melanosporum_Mel28_LAMP  ACATTATTAGGGCCTTGGTAGTTCCTGATTGCATAGCAGATGGGAGGCTGGTCTGAATAGTAGGTTCGAGCTCAGAGCAAGAAGGGATGTATATTGATTTGATAAACCGCATACTCGGTTTCAATATTTATGATTGTTATGGTGCTCCTTGTCAGGTAGTCTTTAATAATAGGAAATGATCCTCACCTACTATGCCCCACCTGTCACGTCCCCCGCTGAGACGTGCTCACCAACTATGGACATGTGAATATAGTCAAATTGGCCCACGGCCTTCCCGTAGCCAAAGGGTCGAATCCAAGCTCCAGTGTACAGTGATAAAATCATCCCGAGTTCCCTCAATGGTGGCACGAGATGCCAATACCGGGATCATGGCATTGGCGTGGAGCACAGAAGCTAAATGCAGAATCACATCAAGGGAAGATGTCGAAGGCGTCACGCATGTACGTCATAGAGGACATCCAATGCATGATACAGCGTCCCCCCACAAACGGAGGATAAATCTCAAACTTGGAAGTGGTCAAAACGTTCACTTCCTGAGTTGGCTATTGTGAATAGGTTGACGGGTATCCGAGTTAATTACTACCCAAATGTCTATACCTATTAACATTCGTGCAATCCTTCTCAAAGGTGGGGTACAATACTGAAGCAAGCTGGCAGTTGCCGCTCGCGAGGTATGAATGCATACTTGTGCTTTGATCATGCAATGTTGGGTTGGTCAGTCATTCGGAGGGCAGGATCTCACAATCAAACCATGCCCAGCTATAAGGGCCATTTACCACTTTTCTTCGTACCAAGGTCTGTGAGGCTGGCATCCGGTAACAGCAAAAGGGTACTAACTCACATACTGAAAGGTAGTCACATGCTGAGGGGTTCTCATTATTTGGCAGCGTGTCACGGGGTCCA |
